# Supplementary material for: Anywhere but here: local conditions motivate dispersal in Daphnia
Source: PeerJ. 2019 Mar 12;7:e6599. doi: 10.7717/peerj.6599 (PMC6419717; doi:10.7717/peerj.6599)
Supplement: Supplemental Information 1 — Test results for differences in the proportion of D. carinata dispersing after 96 hours depending on food availability in patch 1 and population density. A generalised linear model was used with parameter estimates on the logit scale and quasibinomial errors. Whilst patch 1 food availability was found to be significant (P < 0.05), neither the interaction between patch 1 food availability and density (P = 0.404) nor density itself (P = 0.221) were found to be significant. [file peerj-07-6599-s001.pdf]

Table A1: Statistical results for proportion of individuals dispersing after 96 hours of dispersal versus population density. Test results for differences in the proportion of *D. carinata* dispersing after 96 hours depending on food availability in patch 1 and population density. A generalised linear model was used with parameter estimates on the logit scale and quasibinomial errors. Whilst patch 1 food availability was found to be significant ($P < 0.05$), neither the interaction between patch 1 food availability and density ($P = 0.404$) nor density itself ($P = 0.221$) were found to be significant.

Parameter	Estimate (SE)	t stat	P value
Intercept	-1.534 (0.490)	3.128	<0.01
Food available in patch 1	-4.612 (1.612)	2.861	<0.05
Density	0.00564 (0.00443)	1.273	0.221
Food available in patch 1 × *Density*	0.00714 (0.00834)	0.856	0.404
