# Supplementary material for: Anywhere but here: local conditions motivate dispersal in Daphnia
Source: PeerJ. 2019 Mar 12;7:e6599. doi: 10.7717/peerj.6599 (PMC6419717; doi:10.7717/peerj.6599)
Supplement: Supplemental Information 2 — Test results for differences in the proportion of D. carinata juveniles in patch 1 and 2 after 96 hours. A generalised linear model was used with parameter estimates on the logit scale and binomial errors. No effect of patch was found (P = 0.331), suggesting that juveniles were not over-represented amongst dispersers. [file peerj-07-6599-s002.pdf]

Table A2: Statistical results for proportion of juveniles in patch 1 versus patch 2. Test results for differences in the proportion of *D. carinata* juveniles in patch 1 and 2 after 96 hours. A generalised linear model was used with parameter estimates on the logit scale and binomial errors. No effect of patch was found ($P = 0.331$), suggesting that juveniles were not over-represented amongst dispersers.

Parameter	Estimate (SE)	z stat	P value
Intercept	-0.215 (0.103)	2.068	$<$0.05
Patch	0.0865 (0.0889)	0.872	0.331
