# Supplementary material for: Anywhere but here: local conditions motivate dispersal in Daphnia
Source: PeerJ. 2019 Mar 12;7:e6599. doi: 10.7717/peerj.6599 (PMC6419717; doi:10.7717/peerj.6599)
Supplement: Supplemental Information 3 — The effect of population density on the proportion of dispersers after 96 hours, both with and without food available in patch 1 (n = 10 container pairs per treatment). Although higher densities appeared to result in higher proportions of dispersers in both groups, particularly when patch 1 was deprived of food, no significant effect of density was found (Table A1). [file peerj-07-6599-s003.pdf]

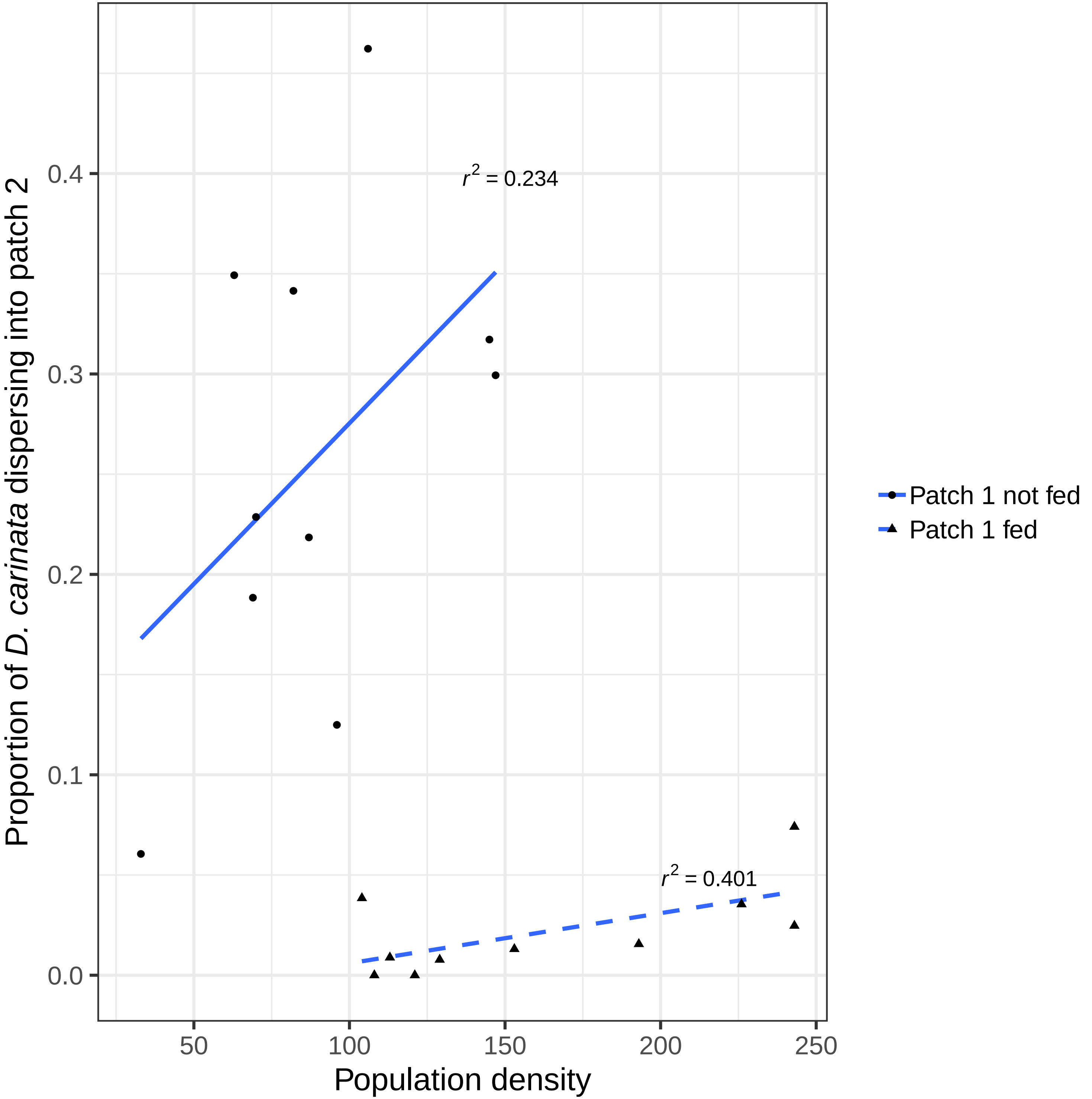

$r^2 = 0.234$
$r^2 = 0.401$
0.4
0.3
0.2
0.1
0.0
50
100
150
200
250
Population density
Proportion of *D. carinata* dispersing into patch 2
Patch 1 not fed
Patch 1 fed
