# Supplementary figures and images for: Anywhere but here: local conditions motivate dispersal in Daphnia
Source: PeerJ. 2019 Mar 12;7:e6599. doi: 10.7717/peerj.6599 (PMC6419717; doi:10.7717/peerj.6599)

Mean patch 2 population size

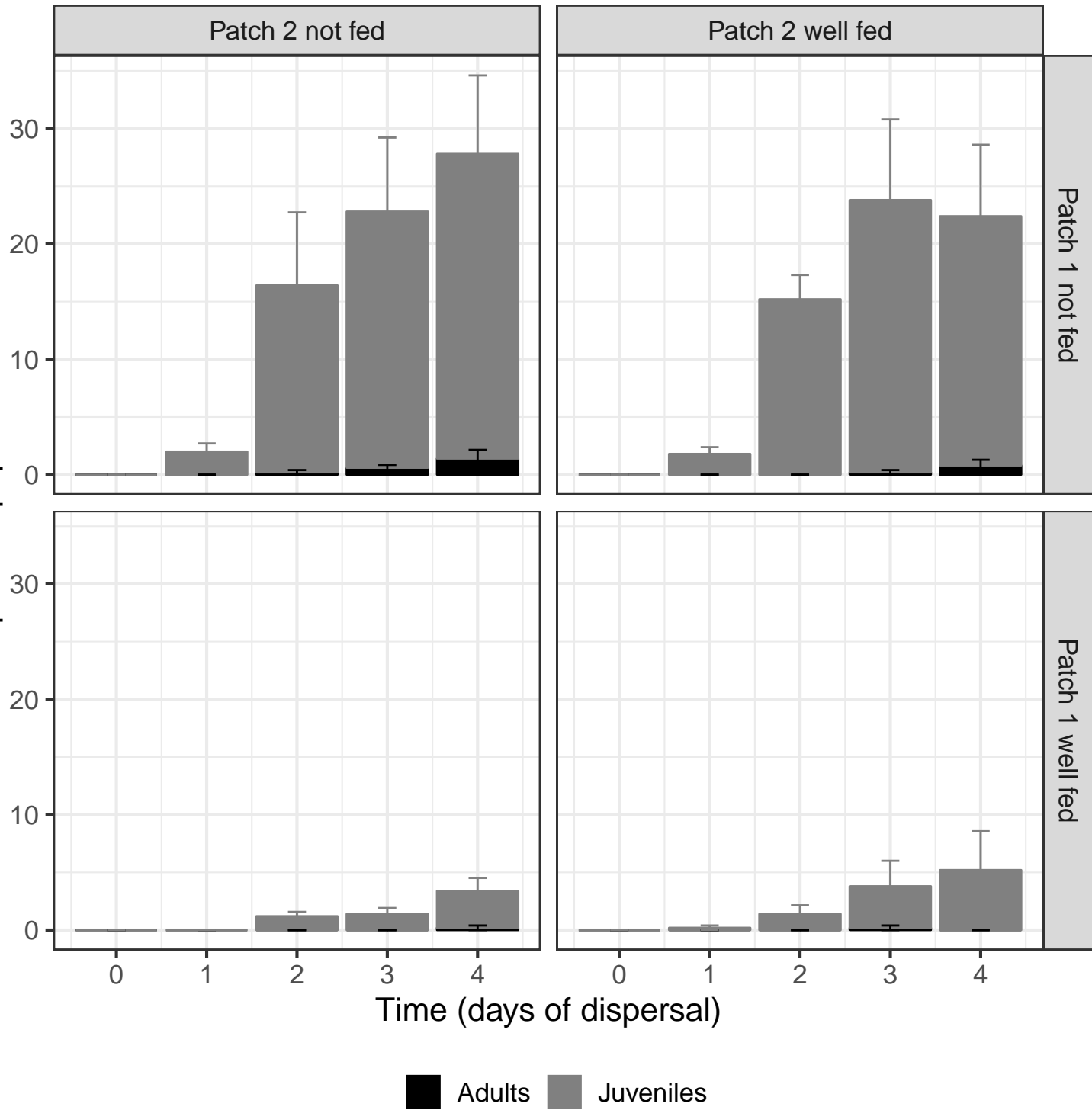

Supplement: Supplemental Information 4 — The effect of feeding regime on mean population size in patch 2 over the dispersal phase (n = 5 container pairs per treatment combination), subdivided by age class. Bars are stacked, such that both adult (black) and juvenile (grey) population sizes combine to indicate mean patch 2 population size. Error bars show SE for each age class’ mean patch 2 population size, rather than for stacked mean patch 2 population size. [file peerj-07-6599-s004.pdf]
